# Surgical Anatomy

**Published:** 1850-01

**Authors:** 


*Surgical Anatomy.* By John Maclise, Surgeon. Part 1. Lea & Blanchard, Philadelphia, 1850. Quarto, pp. 40, with 16 colored plates.

This is an admirable reprint of a deservedly popular London publication. Its English prototype, although not yet completed, has already won its way, amongst our British brethren, to a remarkable success.

Mr. Maclise was previously known as the joint editor of a large work on the Surgical Anatomy of the Arteries, and as the author of a rather transcendental volume on Comparative Osteology; but his last production has evidently been the labor of his especial love. It is the one, at all events, upon which he may safely rest his fame as a most accomplished teacher of a branch of anatomical demonstration, which, whether in its medical or surgical relations, still needs much more elucidation than it generally receives in the systematic courses of the schools. His rare talents as an artist, combined with his attainments as a demonstrator and a surgeon, have enabled him to present a series of illustrations, which, as a whole, are decidedly superior to any that have hitherto appeared.

In guarantee of the anatomical accuracy of these illustrations, their author tells us in his preface, that they were made by himself, from his "own dissections, first planned at the London University College, and afterwards realized at the Ecole Pratique, and School of Anatomy adjoining the Hospital La Pitié, Paris."

With such opportunities and qualifications, we are not surprised at the result in his hands. His artist's eye and mind seem to have aided his surgical ideas, in suggesting a material improvement in the mode of exhibiting the relative anatomy of different parts. We give his own brief account of this improvement in another extract from the preface.

"The unbroken surface of the human figure, is as a map to the surgeon, explanatory of the anatomy arranged beneath; and I have therefore left appended to the dissected regions as much of the undissected as was necessary. My object was to indicate the interior through the superficies, and thereby illustrate the whole living body which concerns surgery, through its dissected dead counterfeit."

Instead, in other words, of two or three confused and complicated displays of a difficult region, such as we find in the works of Velpeau, Blandin, Morton and others, he gives us, in a succession of views, the different stages of the demonstration, retaining, at the same time, as much of the undissected frame as may be needed, to make the whole intelligible at a glance, to the practised student. We have thus an opportunity of comparing, not only the superficial with the deep seated anatomy, but, what is often quite as important although less frequently thought of, the anatomy of the exterior form with that of the regions which it conceals from view. In short, we are happy to remark that the whole study of physical exploration, in its medical as well as surgical relations, is treated in a manner which must essentially facilitate the progress of any one who will take the trouble to verify his own dissections with a careful examination of these plates.

The English copy was issued in folio fasciculi, each containing four plates and eight pages of commentaries and descriptions.

The American is a very elegant and faithful imitation of its London original, in every thing but the form. This, for the sake of convenience, has been changed into a quarto, to be published in four parts, each containing sixteen figures on separate plates, and sixty-four pages of handsome letter-press.

The first two plates are devoted to the form of the thoracic cavity, and the position of the lungs, heart and larger blood-vessels. Plates three and four, exhibit the surgical form of the superficial cervical and facial regions, and the relative positions of the principal blood-vessels, nerves, &c. The third two present us with the deep cervical and facial regions, with their principal vessels, nerves, &c. in relative position. In the fourth two are shown the surgical dissection of the sub-clavian and carotid regions, and the relative anatomy of their contents. Plates nine and ten display the sterno-clavicular or tracheal region, with the relative position of vessels, nerves, &c. In eleven and twelve we have the axillary and brachial regions; in thirteen and fourteen, comparative views of the male and female axillæ; and in plates fifteen and sixteen, the part concludes with the relative anatomy of the bend of the elbow and the forearm.

The commentaries are generally clear and to the purpose, and afford many useful surgical suggestions, although they are unhappily disfigured here and there with some peculiarities of style and language which savor strongly of the author's transcendental predilections. Still it is to be remembered, that the book is essentially one of illustration, and that, as such, it has little to fear from immaterial shortcomings in its literary matter, so long as its plates can boast a superiority that places them almost beyond the reach of competition.

We are too well pleased to enjoy them in their native garb, to quarrel with the few minor whims of phraseology with which they may happen to be burthened. And we feel too thankful to the Philadelphia publishers for their very handsome reproduction of the whole work, and at a rate within every body's reach, not to urge all our medical friends to give it, for their own sakes, the cordial welcome it deserves, in a speedy and extensive circulation.

